# The “Lower Is Better” Debate in Hypertension Management: A Narrative Review of Divergent Recommendations in the 2024 ESC and 2025 ACC/AHA Guidelines

**DOI:** 10.7759/cureus.105433

**Published:** 2026-03-18

**Authors:** Vasudeva Vijaykumar

**Affiliations:** 1 Internal Medicine, Independent Research, Mysore, IND

**Keywords:** acc/aha 2025 guideline, esc 2024 guideline, hypertension, intensive blood pressure control, sprint trial, step trial, systolic blood pressure targets

## Abstract

Hypertension remains a leading modifiable risk factor for cardiovascular disease, standing as a universal health burden that drives immense population-level morbidity and premature mortality. Despite this shared burden, updates to the final published 2024 European Society of Cardiology (ESC) and 2025 American College of Cardiology/American Heart Association (ACC/AHA) clinical practice guidelines have highlighted substantial differences in recommended diagnostic thresholds and treatment targets. This narrative review examines the methodological and interpretative differences between these two major frameworks. It draws on foundational evidence from landmark randomized controlled trials across diverse populations, including older adults, individuals with diabetes, and patients with cerebrovascular disease, to evaluate how the same core evidence base translates into divergent recommendations. Available trial data demonstrate that intensive blood pressure lowering reduces specific cardiovascular outcome domains (such as stroke, heart failure, and cardiovascular mortality) in selected populations but simultaneously increases the risk of treatment-emergent adverse events in others. Ultimately, rather than presenting fundamentally discordant evidence, the differences between the current ESC and ACC/AHA guidelines reflect contrasting committee emphases: the ACC/AHA prioritizes aggregate population-level risk reduction to advocate for a universal <130/80 mm Hg target, whereas the ESC emphasizes subgroup safety and tolerability to justify risk-stratified, individualized treatment ranges.

## Introduction and background

Affecting over a billion adults globally, hypertension constitutes a major modifiable cardiovascular risk factor [1-3]. Contemporary international clinical practice guidelines, including those issued by the European Society of Cardiology (ESC), the American College of Cardiology and American Heart Association (ACC/AHA), and the UK National Institute for Health and Care Excellence (NICE), provide structured recommendations for the diagnosis and management of elevated blood pressure in adults [1,2,4-6]. Although multiple frameworks exist globally, this review focuses specifically on the recent updates to the ESC and ACC/AHA guidelines. Optimizing blood pressure targets is critical because even modest reductions can significantly decrease cardiovascular morbidity and mortality on a population scale; in practice, these guidelines dictate who receives pharmacologic therapy, the intensity of their regimens, and the ultimate balance between preventing severe cardiovascular events and exposing patients to treatment-related adverse events.

To navigate this clinical debate, key terms must be contextualized. Clinical trials routinely compare "intensive" blood pressure control (typically targeting a systolic blood pressure (SBP) of <120 or <130 mm Hg) against "standard" control (typically targeting an SBP of <140 mm Hg) to determine the optimal safety balance [3,7]. Furthermore, guidelines increasingly tailor recommendations based on "patient selection" criteria, such as the presence of "hypertension-mediated organ damage" (HMOD), subclinical structural changes indicating high cardiovascular risk, or "frailty," a state of increased physiological vulnerability that heavily influences a patient's ability to tolerate aggressive antihypertensive regimens [2].

Recent updates to the 2024 ESC and 2025 ACC/AHA guidelines have highlighted substantial differences regarding whether "lower blood pressure is always better." Importantly, both guidelines agree that lower blood pressure reduces cardiovascular risk and strongly advocate individualized care; they differ, however, in three distinct domains: their diagnostic definitions of hypertension, the criteria for initiating pharmacologic treatment, and the framing of recommended SBP targets [1,2]. Their divergence stems from how shared trial evidence is translated into formal recommendations: the ACC/AHA framework prioritizes aggregate population-level risk reduction to justify a universal intensive target, whereas the ESC framework places a stronger emphasis on treatment tolerability and the avoidance of adverse events in vulnerable populations to justify a primary target range [1,2].

These divergent recommendations have emerged despite reliance on a largely overlapping body of randomized controlled trials and evidence syntheses, including large outcome trials evaluating intensive versus standard blood pressure control across diverse populations, such as Systolic Blood Pressure Intervention Trial (SPRINT), Action to Control Cardiovascular Risk in Diabetes Blood Pressure (ACCORD-BP), Strategy of Blood Pressure Intervention in the Elderly Hypertensive Patients (STEP), Secondary Prevention of Small Subcortical Strokes (SPS3), and Hypertension in the Very Elderly Trial (HYVET), as well as multiple post hoc analyses and meta-analyses derived from these datasets [3,7-13].

Contemporary blood pressure targets are derived from these randomized controlled trials by directly comparing the rates of cardiovascular, cerebrovascular, renal, and mortality outcomes between intensive and standard SBP groups [1,2]. Within this shared evidence base, each major trial studied distinct populations. The SPRINT, which studied high-risk adults but notably excluded patients with diabetes mellitus and prior stroke, demonstrated clear cardiovascular benefits with intensive lowering, but simultaneously highlighted trade-offs regarding severe adverse events (specifically acute kidney injury, syncope, and symptomatic hypotension) that prompt divergent guideline interpretations [3,14-16]. Conversely, the ACCORD-BP trial found that the same intensive target in patients with type 2 diabetes did not significantly reduce the primary composite cardiovascular outcome [9]. In older populations, the STEP trial (adults aged 60-80 years) showed substantial cardiovascular benefits with intensive targets. In contrast, the HYVET trial (adults aged ≥80 years) demonstrated that a more moderate target effectively reduced mortality in the very elderly and frail [7,8,12]. Finally, the SPS3 trial evaluated patients with recent lacunar stroke, finding that intensive targets markedly reduced intracerebral hemorrhage without significantly reducing overall recurrent strokes [13].

Furthermore, the contrasting populations in STEP and HYVET highlighted that chronological age alone may not fully dictate cardiovascular risk as much as frailty, a state of decreased physiologic reserve that heavily influences treatment tolerability [2,17].

To synthesize these individual trials into broader clinical recommendations, large-scale evidence syntheses evaluate the aggregate cardiovascular benefit. An individual participant-level meta-analysis conducted by the Blood Pressure Lowering Treatment Trialists' Collaboration, utilizing an intention-to-treat analytic framework rather than achieved blood pressure reductions, reported proportional reductions in major cardiovascular outcomes with pharmacological blood pressure lowering across a wide range of baseline SBP values [18]. However, these proportional benefits must be interpreted cautiously in the absence of explicit adjustment for adverse event burden and competing risk profiles [15,16]. Additional meta-analyses focusing on older adults and individuals with type 2 diabetes mellitus have similarly evaluated intensive versus standard blood pressure control, reporting modest cardiovascular benefits alongside increased risks of specific adverse events, namely hypotension and syncope [10,19].

Post hoc analyses and subgroup analyses from major trials have added further nuance by examining how treatment effects vary according to safety profiles, frailty, and the presence of hypertension-mediated organ damage (such as left ventricular hypertrophy) [15,16,20]. While individualized net-benefit analyses have attempted to quantify the specific trade-offs of intensive lowering within these subgroups, their reliance on post hoc predictive modeling means their findings remain exploratory [15,16].

A major source of differing interpretations of evidence between the two guidelines concerns the generalizability of intensive treatment strategies, with a heavy focus on blood pressure measurement techniques. In particular, the use of unattended automated office measurements in trials such as SPRINT sparked debate over whether its intensive targets could translate to routine clinical practice, as unattended automated measurements can yield systolic readings 5 to 10 mm Hg lower than conventional attended office readings [2,21]. Historical guideline iterations, such as the transition from the JNC 7 report to the 2017 ACC/AHA guideline, documented a gradual evolution toward lower diagnostic thresholds and treatment goals, making these measurement discrepancies increasingly clinically relevant [5,11].

Beyond trial populations, guideline documents and narrative reviews have emphasized the importance of individualized blood pressure management in specific clinical contexts, including advanced age and prior stroke, reflecting concerns related to tolerability and safety [2,13,17]. Against this background, the coexistence of broadly similar evidence bases alongside differing guideline recommendations has renewed debate regarding the principle that "lower is better" for blood pressure control. Importantly, both guidelines firmly agree that lower blood pressure reduces aggregate cardiovascular risk; the debate centers entirely on whether those population-level benefits universally outweigh the individual-level risks of treatment-emergent adverse events [1,2]. This ongoing tension provides the rationale for the present narrative review comparing the 2024 ESC and 2025 ACC/AHA hypertension guidelines.

Methods

This narrative review examined the divergent clinical philosophies of the 2024 ESC and 2025 ACC/AHA hypertension guidelines regarding intensive blood pressure lowering. A targeted literature search of the PubMed database was performed to identify relevant English-language articles published between January 2008 and February 2025. Search terms included "hypertension," "blood pressure target," "intensive blood pressure lowering," and "clinical practice guidelines." Source selection prioritized the foundational evidence base explicitly cited by both guideline committees, primarily comprising landmark randomized controlled trials (e.g., SPRINT, STEP, ACCORD-BP, SPS3, and HYVET) and individual participant-level data (IPD) meta-analyses (e.g., BPLTTC). Select international guidelines (e.g., NICE) and recent post hoc safety evaluations were included to provide external benchmarking and contemporary context.

Rather than performing a de novo risk-of-bias assessment, this review leveraged the independent Evidence Review Committee reports and rigorous methodological grading frameworks already conducted by the ESC and ACC/AHA. Study quality and limitations were evaluated strictly through the lens of how each committee weighed these factors in formulating its guidelines.

Information was synthesized qualitatively using a structured comparative framework. The assigned Class of Recommendation (COR) and Level of Evidence (LOE) from both guidelines were mapped against the primary cardiovascular efficacy endpoints and serious adverse event profiles reported in the shared source trials. This allowed for an analytical comparison of how each committee weighed the trade-off between maximizing population-level cardiovascular risk reduction and mitigating individual-level harms associated with treatment. Because the objective of this review was to analyze clinical policy translation rather than to recalculate previously established statistical models, no quantitative meta-analysis was performed; furthermore, recent comprehensive IPD meta-analyses have already definitively quantified the proportional cardiovascular benefits of blood pressure reduction.

## Review

Guideline recommendations and treatment targets

Both the 2024 ESC and 2025 ACC/AHA guidelines strongly advocate cardiovascular risk stratification, shared decision-making, and individualized care, directly refuting the premise that either framework ignores patient-specific contexts [1,2]. However, they operationalize this individualization differently across three conceptually distinct domains: diagnostic thresholds, treatment initiation, and achieved treatment goals. The ESC guideline defines hypertension using the diagnostic threshold of ≥140/90 mm Hg and recommends a structured target range of 120-129 mm Hg for most adults [2]. Crucially, the ESC advises individualizing this achieved target goal based on vulnerabilities, explicitly adopting the ALARA ("As Low As Reasonably Achievable") principle to allow more lenient targets (e.g., <140/90 mm Hg) for frail patients, older adults (aged ≥85 years), and those with symptomatic orthostatic hypotension to prioritize safety over maximum theoretical benefit [2,17].

In contrast, the 2025 ACC/AHA guideline defines hypertension using a lower diagnostic threshold of ≥130/80 mm Hg [1]. To individualize treatment initiation, the ACC/AHA employs the PREVENT (Predicting Risk of Cardiovascular Disease EVENTs) equations to quantify 10-year risk; however, once the clinical decision to initiate pharmacotherapy is made, the guideline endorses a universal intensive blood pressure target of <130/80 mm Hg for the vast majority of adults [1]. This guideline builds on prior 2017 ACC/AHA recommendations by reinforcing the role of intensive blood pressure lowering as the primary strategy to reduce major cardiovascular events, while explicitly acknowledging that clinical judgment and shared decision-making remain necessary in populations at high risk of treatment-related adverse effects [1,5].

Ultimately, the differences between these contemporary guidelines reflect distinct interpretations of shared trial evidence, as reflected in the differing COR and LOE assigned by each committee to the same data to manage the benefit-harm balance [1,2]. The ESC guideline explicitly integrates safety findings from randomized trials, meta-analyses, and post hoc analyses to assign strong recommendations for target ranges and specific contraindications (e.g., Class IIa/IIb recommendations for lenient targets), supporting a cautious, patient-centered approach for individuals with multimorbidity [2].

Conversely, the ACC/AHA guideline places greater emphasis on the aggregate cardiovascular risk reduction observed in large outcome trials and pooled analyses to assign a Class 1 recommendation for a universal goal of <130/80 mm Hg [1]. The principal structural and diagnostic differences between the 2024 ESC and 2025 ACC/AHA hypertension guidelines are summarized in Table 1.

**Table 1:** Key Differences Between the 2024 ESC and 2025 ACC/AHA Hypertension Guidelines ALARA: As Low As Reasonably Achievable; BP: Blood Pressure; CKD: Chronic Kidney Disease; CVD: Cardiovascular Disease; HMOD: Hypertension-Mediated Organ Damage; PREVENT: Predicting Risk of cardiovascular disease EVENTs; SCORE2-OP: Systematic Coronary Risk Evaluation 2–Older Persons.

Feature	2024 ESC Guideline [2]	2025 ACC/AHA Guideline [1]
Definition of Hypertension	Office BP ≥140/90 mm Hg (Class I, Level B). Elevated BP: 120–139/70–89 mm Hg (new category; not defined as hypertension).	Office BP ≥130/80 mm Hg (Class 1, Level B-NR). Elevated BP: 120–129/<80 mm Hg. Stage 1: 130–139/80–89 mm Hg. Stage 2: ≥140/90 mm Hg.
CV Risk Assessment Tool	SCORE2/SCORE2-OP. Estimates the 10-year risk of fatal and non-fatal CVD events (MI, stroke). Calibrated for four distinct European risk regions.	PREVENT™ (Predicting Risk of CVD EVENTs); Estimates the 10-year risk of CVD (including heart failure). Incorporates kidney function and social deprivation index; replaces pooled cohort equations.
Pharmacotherapy Initiation	Risk-Based Strategy: BP ≥140/90: Immediate drug treatment for all (Class I, Level A). BP 130–139/80–89: Drug treatment only if sufficiently high risk (established CVD, Diabetes, moderate/severe CKD, HMOD, or 10-year CVD risk ≥10%) after a three-month trial of lifestyle intervention (Class I, Level A).	Risk-Based Strategy: BP ≥140/90: Immediate drug treatment for all (Class 1, Level A). BP 130–139/80–89: Immediate drug treatment if high risk (CVD, Diabetes, CKD, or 10-year CVD risk ≥7.5%) (Class 1, Level A and Class 1, Level C-LD). Low Risk (<7.5%): Drug treatment only after a 3–6 month trial of lifestyle intervention fails (Class 1, Level B-R).
Primary Systolic Target	Systolic 120–129 mm Hg (Class I, Level A). Recommended for most adults receiving treatment, provided it is well tolerated. Emphasizes a target range to avoid hypotension.	<130 mm Hg (Class 1, Level A) Recommended for adults with hypertension and increased CVD risk. Focuses on an upper limit threshold (with <120 mm Hg recommended when feasible).
Diastolic Target	Diastolic 70–79 mm Hg (Class IIb, Level C) may be considered as the ideal on-treatment range if systolic BP is controlled but diastolic remains ≥80 mm Hg, to avoid isolated diastolic hypertension.	<80 mm Hg (Class 1, Level B-R). Recommended for adults at increased CVD risk (≥7.5%). <80 mm Hg (Class 2b, Level B-NR). May be reasonable for adults not at increased CVD risk (<7.5%).
Management of Frail/Older Adults	"ALARA" Principle: For frail patients, those aged ≥85 years, or those with symptomatic orthostatic hypotension, the threshold to consider drug treatment is modified to ≥140/90 mm Hg (Class IIa, Level B). In these groups, the guideline formally recommends targeting a blood pressure that is "As Low As Reasonably Achievable" (Class I, Level A), utilizing more lenient goals (e.g., <140/90 mm Hg) to prioritize tolerability over strict numeric control [Class IIa/IIb, Level C].	Clinical Judgment: For adults aged ≥80 years, initiation at ≥130/80 mm Hg is recommended if the benefits outweigh the harms. Emphasizes shared decision-making; chronological age alone is not a barrier to intensive control.
Emphasis on Safety & Tolerability	Tolerability First: Explicitly notes that trial conditions differ from routine care. Advises relaxing targets if orthostatic hypotension is present. Recommends deprescribing if BP drops due to progressing frailty.	Risk Reduction Priority: Notes that adverse events in trials were "infrequent and usually mild." Cites evidence from randomized trials demonstrating that intensive BP lowering does not increase the risk of orthostatic hypotension or falls. Emphasizes careful monitoring rather than raising targets.

Rather than a sudden paradigm shift, this contemporary divergence is the direct methodological culmination of decisions made in preceding guideline cycles [5,6,11]. Specifically, the 2017 ACC/AHA guideline and its supporting systematic review fundamentally restructured American clinical practice by officially lowering the diagnostic threshold to ≥130/80 mm Hg [5,11]. Conversely, while the 2018 ESC/ESH guideline also aimed for lower treatment targets in fit patients, the European committee deliberately maintained the traditional ≥140/90 mm Hg diagnostic threshold, adopting explicit cautions regarding tolerability and adverse events in older populations [6]. This historical split regarding the definition of the disease itself established the exact structural and philosophical precedent for the differing treatment targets observed in the 2024 and 2025 updates.

Interpretation of randomized controlled trials 

Randomized controlled trials comparing intensive and standard blood pressure targets constitute the foundational evidence base informing both European and North American guidelines [1,2]. The landmark SPRINT enrolled high-risk adults without diabetes or prior stroke and achieved a mean SBP of 121.4 mm Hg in the intensive group versus 136.2 mm Hg in the standard group [3]. While SPRINT demonstrated a significant reduction in major cardiovascular events and all-cause mortality, it also reported higher rates of acute kidney injury and hypotension [3,14].

Furthermore, a prespecified subgroup analysis of SPRINT demonstrated that these intensive targets yielded significant cardiovascular and mortality benefits, even in adults aged ≥75 years, providing the empirical foundation for applying these targets to older adults [22]. To address these findings and implement them into clinical practice, the 2025 ACC/AHA committee prioritized the cardiovascular mortality benefit to assign a Class 1 recommendation for a universal <130/80 mm Hg target, whereas the 2024 ESC committee explicitly cited the adverse events to justify careful tolerability monitoring and higher target ranges in frail adults [1,2].

Subsequent secondary analyses of SPRINT data have explored the heterogeneity of these treatment effects to inform clinical applications better. Specifically, patient-level evaluations using individualized predictive modeling have examined the balance between cardiovascular benefit and treatment-related harms, highlighting variability in net benefit according to baseline risk, age, and susceptibility to adverse events [15,16]. Reflecting these nuances, the ESC guideline explicitly incorporates these clinical vulnerabilities into its framework to formulate its Class IIa and IIb recommendations, which allow for less stringent target ranges in populations prone to polypharmacy and frailty [2,15,16].

To evaluate target applicability in specific populations, the ACCORD-BP trial specifically enrolled patients with type 2 diabetes [9]. Achieving a mean systolic pressure of 119.3 mm Hg in the intensive group versus 133.5 mm Hg in the standard group, ACCORD-BP did not demonstrate a significant reduction in its primary composite cardiovascular outcome [9]. The 2024 ESC guideline explicitly cites this lack of primary composite benefit to support its Class I recommendation for a more cautious 120-129 mm Hg target range in diabetic patients, contrasting directly with the ACC/AHA's endorsement of a uniform <130/80 mm Hg threshold [1,2,9].

Guideline target recommendations are further shaped by the inclusion criteria of age- and disease-specific trials. For instance, the 2025 ACC/AHA guideline heavily relies on the STEP trial, which enrolled patients aged 60 to 80 years-to formally justify its intensive <130 mm Hg target recommendation in older adults, given its finding of reduced cardiovascular events with intensive control [1,7,12]. Conversely, for cerebrovascular disease, the SPS3 trial investigated lower targets (<130 mm Hg) in patients with recent lacunar stroke [13]. While earlier evidence notes that intensive lowering generally reduces stroke risk, SPS3 found no significant reduction in the overall risk of recurrent stroke (which includes ischemic events); however, both the ESC and ACC/AHA highlight its significant reduction in the specific risk of intracerebral hemorrhage to justify secondary stroke prevention targets [1,2,13].

Finally, the HYVET demonstrated that among patients aged ≥80 years with a remarkably high baseline SBP (mean ~173 mm Hg), targeting a moderate reduction to <150/80 mm Hg effectively reduced the risk of stroke and all-cause mortality [8]. These distinct inclusion criteria and high baseline risks continue to inform ESC discussions regarding the safety of overly aggressive targets in very elderly and frail populations [2]. The key design characteristics, SBP targets, and principal findings of these landmark trials are summarized in Table 2.

**Table 2:** Major Randomized Trials Comparing Intensive and Standard Blood Pressure Targets AKI: acute kidney injury; BP: blood pressure; SBP: systolic blood pressure; SPRINT: Systolic Blood Pressure Intervention Trial; STEP: Strategy of Blood Pressure Intervention in the Elderly Hypertensive Patients; SPS3: Secondary Prevention of Small Subcortical Strokes; HYVET: Hypertension in the Very Elderly Trial.

Trial	Age Range	Number of Participants (n)	Population & Key Exclusions	SBP Target (Intensive vs. Standard)	Achieved Mean SBP (Intensive vs. Standard)	Principal Findings & Interpretation Stated by Trial Authors
SPRINT	≥50 years	9,361	High-risk adults without diabetes. (Excluded: diabetes, prior stroke, institutionalized).	<120 mm Hg Vs <140 mm Hg	121.4 mm Hg Vs 136.2 mm Hg	Intensive SBP lowering significantly reduced rates of major cardiovascular events and all-cause mortality, but was associated with higher rates of selected adverse events (AKI, hypotension, syncope), indicating benefit with increased treatment-related risk [3].
SPRINT (Subgroup)	≥75 years	2,636	Pre-specified subgroup of SPRINT participants aged ≥75 years.	<120 mm Hg vs <140 mm Hg	123.4 mm Hg vs 134.8 mm Hg	Intensive SBP control in this older demographic significantly reduced the rates of major cardiovascular events and all-cause mortality, supporting intensive targets in older adults [22].
ACCORD-BP	40–79 years	4,733	Adults with type 2 diabetes at high cardiovascular risk.	<120 mm Hg Vs <140 mm Hg	119.3 mm Hg Vs 133.5 mm Hg	Intensive BP control did not significantly reduce the primary composite cardiovascular outcome or all-cause mortality compared with standard control, although stroke incidence was significantly lower [9].
STEP	60–80 years	8,511	Older Chinese adults with hypertension. (Excluded: prior stroke, severe frailty).	110–130 mm Hg Vs 130–150 mm Hg	127.5 mm Hg Vs 135.3 mm Hg	Targeting lower systolic blood pressure significantly reduced the incidence of cardiovascular events in older adults compared with standard treatment. Incidence of hypotension was higher in the intensive group [7].
SPS3	≥30 years	3,020	Patients with recent lacunar stroke. (Excluded: cortical ischemic stroke).	<130 mm Hg Vs 130–149 mm Hg	127 mm Hg Vs 138 mm Hg	Intensive targets did not significantly reduce the primary outcome of all recurrent stroke (including ischemic), but significantly reduced the specific risk of intracerebral hemorrhage [13].
HYVET	≥80 years	3,845	Very elderly adults with hypertension. (Baseline SBP was extremely high: ~173 mm Hg).	Target <150/80 mm Hg Vs Placebo	~143 mm Hg Vs ~158 mm Hg	Antihypertensive treatment leading to moderate blood pressure reduction safely reduced the risk of death from any cause and heart failure, supporting the avoidance of overly aggressive targets in very elderly patients [8].

Evidence from meta-analyses and pooled data

Meta-analyses and pooled analyses have played an important role in shaping contemporary perspectives on intensive blood pressure lowering by synthesizing results across randomized controlled trials with differing populations and target thresholds. An individual participant-level meta-analysis conducted by the Blood Pressure Lowering Treatment Trialists' Collaboration, utilizing an intention-to-treat analytic framework rather than achieved blood pressure reductions, demonstrated proportional reductions in major cardiovascular outcomes with pharmacological blood pressure lowering across a wide range of baseline SBP levels (an approximate 10% relative risk reduction per 5 mm Hg lowering) [18]. Both European and North American committees explicitly cite these findings to agree that the relative cardiovascular benefits of blood pressure reduction are broadly generalizable; however, they diverge in clinical implementation. The 2025 ACC/AHA guideline specifically leverages this proportional risk reduction to justify its universal <130/80 mm Hg target, whereas the 2024 ESC guideline argues that these relative benefits must be weighed against absolute baseline risks and individual tolerability constraints [1,2,18].

Recent subgroup-specific meta-analyses, while not universally cited in the guidelines themselves due to publication timelines, independently synthesize the exact primary trial data (such as SPRINT, STEP, and ACCORD) that the committees evaluated, clearly illustrating the empirical basis for their divergent recommendations. Analyses restricted to older adults have yielded nuanced data; Seidu et al. reported that intensive blood pressure control significantly reduced the risk of major adverse cardiovascular events and cardiovascular mortality in older persons, with no increase in serious adverse events (specifically defined as gastrointestinal and respiratory symptoms, severe hypotension, and abnormal laboratory findings) [23]. Conversely, Li et al. observed that while intensive goals reduced cardiovascular events, they were associated with a significantly increased risk of specific harms, namely hypotension and syncope, reinforcing the need for careful patient selection [10]. Similarly, in individuals with type 2 diabetes mellitus, pooled analyses evaluating intensive versus standard control identified limited cardiovascular benefit beyond stroke reduction [19]. This specific lack of composite benefit independently validates the 2024 ESC guideline's Class I recommendation that cautions against the uniform application of aggressive <120 mm Hg targets in diabetic populations [2,19].

Secondary and post hoc analyses from large trials have complemented this pooled evidence by exploring specific clinical and metabolic subgroups. For instance, age-stratified evaluations of SPRINT demonstrated significant cardiovascular benefits in adults aged ≥75 years, establishing that advanced chronological age alone does not necessarily negate the efficacy of intensive control [22]. Rather than treating older adults as a monolith, contemporary post hoc analyses from the STEP trial examined how treatment effects were modified by specific baseline parameters, including fasting serum glucose and risk of new-onset diabetes, remnant cholesterol levels, and true resistant hypertension status [24-26]. By proving that these specific clinical sub-phenotypes derive varying magnitudes of net benefit from intensive lowering, these emerging analyses directly inform and validate the ESC's emphasis on highly individualized, patient-centered treatment approaches and precision medicine over universal numerical rules [15,16,24-26]. Evidence from meta-analyses, pooled analyses, and post hoc evaluations relevant to intensive blood pressure lowering is summarized in Table 3.

**Table 3:** Meta-Analyses, Pooled Analyses, and Post-Hoc Evidence Evaluating Intensive Blood Pressure Lowering ALARA: As Low As Reasonably Achievable; BP: blood pressure; BPLTTC: Blood Pressure Lowering Treatment Trialists’ Collaboration; MACE: Major Adverse Cardiovascular Events; SBP: systolic blood pressure; STEP: Strategy of Blood Pressure Intervention in the Elderly Hypertensive Patients.

Analysis	Population Included	BP Targets/Ranges Evaluated	Comparator	The Main Finding Stated by the Authors	Guideline Alignment
BPLTTC individual participant–data meta-analysis [18].	Adults from multiple randomized trials	Standardized 5 mm Hg SBP reductions across various baseline strata (<120 to ≥170 mm Hg)	Higher achieved SBP levels	Blood pressure lowering was associated with proportional reductions in major cardiovascular events across a wide range of baseline blood pressure values (~10% relative risk reduction per 5 mm Hg SBP lowering).	Explicitly cited by both; ACC/AHA leverages this for a universal <130 mm Hg target, while ESC balances this relative benefit against absolute baseline risk [1,2,18].
Meta-analysis in older adults	Older adults (≥65 y) with or without diabetes	Intensive targets (ranging from <120 to <140 mm Hg)	Standard targets (ranging from <140 to <160 mm Hg)	Intensive control significantly reduced MACE (I 2 = 0%) and cardiovascular mortality with no increase in the risk of serious adverse events or renal failure.	Reflects the empirical trial data that drove the ACC/AHA to recommend intensive control in older adults based on clinical judgment [1,23].
	Adults >60 y (SPRINT, STEP, ACCORD participants)	Intensive targets (<120 mm Hg or 110–130 mm Hg)	Standard targets (<140 mm Hg or 130–150 mm Hg)	Intensive control reduced MACE (I 2 = 20%) and stroke but did not significantly reduce mortality; it was associated with significantly increased risks of hypotension (I 2 = 44%) and syncope (I 2 = 0%).	Illustrates the exact harm data profiles that drove the ESC to adopt the "ALARA" principle and lenient target ranges for frail older populations [2,10].
Meta-analysis in diabetes (Ioannidou et al.) [19].	Adults with type 2 diabetes mellitus	Variable intensive targets	Variable standard targets	Intensive control significantly reduced the risk of stroke and macro-albuminuria but did not significantly reduce other major cardiovascular outcomes or mortality (I 2 = 41.5%).	Independently validates the ESC's decision to recommend a cautious 120–129 mm Hg target range, diverging from the ACC/AHA's uniform <130 mm Hg goal [2,19].
STEP Long-Term Follow-up [12].	Older adults enrolled in STEP (6-year follow-up)	Sustained Intensive (110–<130 mm Hg)	Delayed Intensive (Transitioned from Standard)	Sustained intensive treatment yielded greater cardiovascular benefits than delayed intensification, with earlier initiation associated with greater risk reduction.	Provides emerging post hoc validation for the ACC/AHA strategy of aggressive early initiation [1,12].
STEP Remnant Cholesterol Analysis [24].	STEP participants stratified by metabolic parameters	Intensive (110–<130 mm Hg)	Standard (130–<150 mm Hg)	Remnant cholesterol modified treatment effects: patients with higher remnant cholesterol derived greater cardiovascular and mortality benefits from intensive BP lowering than those with lower levels.	Validates the ESC’s framework prioritizing patient-level sub-phenotype risk stratification (precision medicine) over uniform numerical rules [2,24].
STEP Resistant Hypertension Analysis [26].	STEP participants with true resistant hypertension	Intensive (110–<130 mm Hg)	Standard (130–<150 mm Hg)	Intensive BP control significantly reduced cardiovascular events in patients with resistant hypertension without increasing the risk of adverse outcomes.	Supports the extension of intensive targets to difficult-to-treat phenotypes, provided polypharmacy is managed safely [26].

Methodological considerations and blood pressure measurement

Methodological factors, particularly blood pressure measurement techniques, have been central to debates regarding the applicability of intensive blood pressure targets derived from randomized controlled trials. Early criticisms suggested that the automated office blood pressure measurements used in the SPRINT generated significantly lower values than conventional measurements, with guideline committees noting that non-standardized routine office readings are typically 5-15 mm Hg higher than standardized trial measurements [6]. However, secondary analyses conducted within SPRINT evaluated the specific use of attended versus unattended measurements and demonstrated that achieved blood pressure levels and cardiovascular risk reductions were highly similar across both approaches (e.g., the mean achieved SBP was 121.0 mm Hg in the attended group versus 121.4 mm Hg in the unattended group) [21]. This established that the critical factor ensuring accuracy is not whether clinic staff are in the room, but rather strict adherence to a standardized protocol involving a quiet rest period and multiple averaged readings [21]. Therefore, the concern that trial findings may not translate to routine clinical practice stems directly from the reality that busy real-world clinics frequently omit these standardized steps, leading to artificially inflated blood pressure readings and a subsequent risk of overtreatment if aggressive targets are applied [2,21].

These methodological realities have been explicitly referenced in guideline discussions, leading the two committees to interpret the same measurement concept differently. The 2025 ACC/AHA guideline mandates that standardized measurement protocols must be employed in clinical practice, utilizing this requirement to confidently justify the broad application of an intensive <130/80 mm Hg target [1]. In contrast, the 2024 ESC guideline explicitly acknowledges the implementation gap, warning that because real-world clinics often fail to utilize strict standardized protocols, applying an aggressive <120 mm Hg trial target to a casual office reading could induce severe hypotension [2]. The ESC uses this methodological caution to justify their recommendation for a more lenient primary target range of 120-129 mm Hg in routine care [2].

Beyond measurement techniques, broader methodological considerations such as trial inclusion criteria and baseline cardiovascular risk heavily influence guideline formulation. While large outcome trials successfully demonstrate the benefits of intensive treatment goals for their enrolled cohorts, they systematically exclude highly vulnerable demographics [3,7]. Rather than extrapolating these intensive targets universally, the ESC guideline directly utilizes these trial exclusions to stress individualized treatment decisions [2]. Consequently, for populations heavily underrepresented in the intensive arms of pivotal trials, such as very elderly individuals (aged ≥85 years), those with severe frailty, and patients with symptomatic orthostatic hypotension, the ESC guideline explicitly pivots toward clinical judgment and individualized leniency (such as targeting <140/90 mm Hg) to prioritize safety over strict numerical control [2].

Safety, adverse events, and individualized benefit-harm assessment

Safety considerations and treatment-related adverse events have been consistently emphasized in evaluations of intensive blood pressure lowering and play a central role in contemporary guideline recommendations [1,2]. The SPRINT reported higher rates of selected adverse events in participants assigned to intensive targets [3]. To accurately contextualize these risks, it is critical to distinguish between severe treatment-emergent events and less severe symptomatic disturbances; SPRINT noted significant increases in serious adverse events (SAEs), defined specifically as those resulting in hospitalization or emergency department evaluation, such as severe hypotension, syncope, electrolyte abnormalities, and acute kidney injury [3,14]. These specific safety profiles have been incorporated into both ESC and ACC/AHA guideline discussions to mandate careful clinical monitoring [1,2].

Subsequent exploratory, patient-level analyses of SPRINT data have examined heterogeneity in treatment effects and quantified individualized net benefit by simultaneously considering cardiovascular risk reduction and adverse event burden [15,16]. Given their reliance on post hoc predictive modeling and simulated preference weights, these analyses are inherently hypothesis-generating [15,16]. Nevertheless, they demonstrated that the balance between benefit and harm varied substantially by baseline cardiovascular risk, age, and vulnerability to adverse events, providing empirical support for ESC guideline recommendations advocating individualized blood pressure targets rather than uniform intensification for all patients [2,15,16].

Evidence from other randomized trials and secondary analyses has reinforced the importance of safety considerations in specific populations, directly shaping how these populations are treated in the guidelines [10,23]. For instance, the prespecified SPRINT subgroup analysis of patients aged ≥75 years demonstrated that while the absolute rates of specific adverse events were higher, the overall rate of serious adverse events did not differ significantly between treatment groups, confirming a robust net cardiovascular benefit in older adults [22]. Alongside evidence from the STEP trial and its long-term follow-up, the 2025 ACC/AHA guideline explicitly incorporates these findings to confidently justify a <130/80 mm Hg target in older populations, provided monitoring is maintained [1,7,12]. Conversely, findings from the ACCORD-BP trial highlighted an increased risk of SAEs without a significant reduction in the primary composite cardiovascular outcome in individuals with type 2 diabetes mellitus [9]. The 2024 ESC guideline incorporates this specific limitation to justify a more cautious, lenient primary target range (120-129 mm Hg) for diabetic patients, in contrast to the AHA's uniform approach [2,9,19].

Post hoc evaluations and narrative reviews have further contextualized safety concerns by examining frailty, comorbidity burden, and secondary outcomes in vulnerable populations, such as very elderly individuals and patients with prior cerebrovascular disease [2,16,17]. For frail older adults or those with limited life expectancy, the 2024 ESC guideline formally adopts the ALARA principle ('As Low As Reasonably Achievable') and explicitly prioritizes tolerability and less stringent targets (e.g., <140/90 mm Hg) over rigid adherence to numeric goals [2]. In stark contrast, the 2025 ACC/AHA guideline maintains a primary focus on absolute risk reduction even in advanced age; it recommends that for adults aged ≥80 years, treatment initiation at ≥130/80 mm Hg should still be pursued based on shared decision-making, arguing that chronological age or frailty alone should not act as a strict barrier to intensive control if clinical judgment suggests the cardiovascular benefits outweigh the harms [1]. Key safety outcomes, adverse events, and individualized benefit-harm assessments from major trials are summarized in Table 4.

**Table 4:** Safety Outcomes, Adverse Events, and Individualized Benefit–Harm Evidence From Intensive Blood Pressure Lowering Trials AE: adverse event; AHA: American Heart Association; ALARA: As Low As Reasonably Achievable; BP: blood pressure; CV: cardiovascular; ED: emergency department; ESC: European Society of Cardiology; SAE: serious adverse event; SBP: systolic blood pressure; SPRINT: Systolic Blood Pressure Intervention Trial; STEP: Strategy of Blood Pressure Intervention in the Elderly Hypertensive Patients; SPS3: Secondary Prevention of Small Subcortical Strokes; HYVET: Hypertension in the Very Elderly Trial.

Study & Population	Intensive SBP Target	Safety Outcomes Assessed	Main Safety-Related Finding Stated by the Authors	Guideline Alignment & Citation Status
SPRINT Primary Analysis [3]. High-risk adults without diabetes	<120 mm Hg	Hypotension, syncope, electrolyte abnormalities, and acute kidney injury.	Intensive lowering was associated with higher rates of serious adverse events (requiring hospitalization or ED visits), particularly severe hypotension and acute kidney injury.	Explicitly cited by both guidelines. Both committees utilize this primary data to mandate standardized monitoring for treatment-emergent harms during titration.
SPRINT Safety & Measurement (Johnson et al.) [21]. SPRINT participants	<120 mm Hg	Serious adverse events and kidney outcomes across measurement types.	Rates of adverse events associated with intensive treatment were consistent across different BP measurement approaches, indicating safety findings were not attributable to measurement technique.	Illustrates AHA Rationale. While guidelines rely on the primary trial, this secondary analysis illustrates the empirical basis for the AHA's mandate that standardized measurement protocols must be used to safely achieve <130 mm Hg.
SPRINT Individualized Net Benefit (Bress et al. / Jamshidian et al.) [15,16]. Stratified by baseline risk	<120 mm Hg	CV benefit versus serious adverse events.	(Exploratory / Post hoc model): The balance of benefit and harm varied by baseline risk; higher-risk (older/frail) individuals incurred more harm but derived a greater absolute net benefit due to greater CV risk reduction.	Validates ESC Strategy. As a post hoc model, it independently validates the empirical foundation for the ESC's strategy of individualizing targets based on baseline frailty rather than universal rules.
SPRINT Older Adult Subgroup (Williamson et al.) [22]. Adults aged ≥75 years	<120 mm Hg	Serious adverse events and tolerability.	Intensive treatment reduced cardiovascular events and mortality without significantly increasing the overall rate of serious adverse events or injurious falls, despite higher absolute rates of specific events like acute kidney injury.	Explicitly cited by AHA. The ACC/AHA utilizes this subgroup safety data to justify that chronological age alone should not act as a barrier to intensive targets, provided shared decision-making is utilized.
STEP Long-Term Follow-up (Song et al.) [12]. Older adults (≥60 y)	110–130 mm Hg	Cardiovascular outcomes and tolerability.	Sustained intensive control provided long-term cardiovascular benefit; hypotension was the only adverse event significantly increased by intensive treatment.	Explicitly cited by AHA. The primary STEP trial is heavily cited by the AHA to formally justify intensive <130 mm Hg targets in older adults. The recent 2025 follow-up provides emerging validation.
ACCORD-BP [9]. Adults with type 2 diabetes	<120 mm Hg	Serious adverse events.	Intensive lowering increased the frequency of treatment-related SAEs without a significant reduction in the primary composite CV outcome.	Explicitly cited by ESC. The ESC directly incorporates this lack of composite benefit and increased harm to justify a cautious 120–129 mm Hg target for diabetics.
SPS3 Trial [13]. Patients with recent lacunar stroke	<130 mm Hg	Serious adverse events and tolerability.	Lower BP targets were safe and well tolerated, and significantly reduced intracerebral hemorrhage, though primary overall stroke reduction was not statistically significant.	Explicitly cited by both guidelines. Both committees cite this safety profile to support intensive targets specifically for the secondary prevention of hemorrhagic stroke.
HYVET [8]. Adults aged ≥80 years with hypertension	Target <150 mm Hg	Serious adverse events.	Antihypertensive treatment leading to moderate BP reduction was well tolerated and reduced stroke and mortality, supporting the avoidance of overly aggressive targets in very elderly patients.	Explicitly cited by ESC. The ESC utilizes this cohort data to validate the ALARA principle ("As Low As Reasonably Achievable") and support lenient <140/90 mm Hg targets in the very elderly.

Global perspectives and guideline synthesis

Beyond European and North American recommendations, international guideline documents provide an external benchmark that contextualizes the ESC-ACC/AHA dichotomy. For example, the UK National Institute for Health and Care Excellence (NICE) guideline recommends a clinic blood pressure target of <140/90 mm Hg for adults under 80 years of age, and a more lenient target of <150/90 mm Hg for adults aged 80 and older [4]. Rather than contrasting with both major societies, the NICE framework explicitly aligns with the ESC's age-stratified, conservative philosophy, standing in stark contrast to the ACC/AHA's universal, intensive <130/80 mm Hg target [1,2,4].

Comparative observational analyses demonstrate how these varying diagnostic thresholds impact clinical classification and risk detection. For instance, a study by de Souza et al. compared the 2017 ACC/AHA and 2018 ESC/ESH criteria in young adults, finding that the lower ACC/AHA thresholds identified a higher prevalence of hypertension and were significantly more accurate at detecting early vascular aging, as measured objectively by increased pulse wave velocity [27]. However, because this analysis relied on cross-sectional population modeling, it inherently lacks longitudinal follow-up, limiting the ability to definitively prove that initiating treatment based on these lower detection thresholds ultimately prevents hard cardiovascular endpoints over time [27].

Collectively, these guidelines, randomized trials, and secondary evaluations underscore that contemporary disagreements regarding optimal blood pressure targets arise not from fundamentally discordant evidence bases, but from philosophical differences in implementation. The core divergence is clear: the ACC/AHA framework prioritizes maximum population-level cardiovascular risk reduction through universal, intensive targets, whereas the ESC prioritizes individual safety, the avoidance of polypharmacy, and strict tolerability in vulnerable subgroups [1,2,18].

Discussion

The present narrative review highlights that the ongoing debate over whether "lower is better" for blood pressure control is rooted less in conflicting data than in fundamentally divergent clinical interpretations of the same evidence base. Both the 2024 ESC and 2025 ACC/AHA guidelines draw their primary recommendations from the same core randomized controlled trials (e.g., SPRINT, STEP, and ACCORD-BP) and meta-analyses [1-3,7,9,18]. The divergence arises from opposing clinical philosophies: the ACC/AHA framework prioritizes maximizing population-wide cardiovascular event reduction, whereas the ESC framework heavily prioritizes individual harm avoidance, tolerability, and the realities of routine clinical implementation.

Randomized trials collectively demonstrate that intensive blood pressure lowering clearly reduces major cardiovascular outcomes in specific populations, such as high-risk adults without diabetes and fit older adults [3,7]. However, these same trials demonstrate an increased risk of treatment-related adverse events, such as severe hypotension and acute kidney injury, in distinct clinical subgroups, notably individuals with type 2 diabetes and those with severe baseline frailty [3,9,15,16]. The ESC guideline places particular emphasis on this clinical heterogeneity. Rather than assigning universal treatment ranges for all high-risk patients, the ESC incorporates these safety analyses to support highly stratified SBP targets, formally recommending more lenient goals (e.g., <140/90 mm Hg) for patients aged ≥85 years, those with multimorbidity, and those at the highest risk for adverse events [2,6].

In contrast, the ACC/AHA guideline framework places far greater weight on the aggregate cardiovascular risk reduction observed in pooled analyses. This includes relying heavily on individual participant-level meta-analyses that demonstrate a proportional cardiovascular benefit across a wide range of baseline blood pressure levels [1,18]. However, as noted in methodological critiques of the guidelines, these pooled efficacy estimates must be interpreted cautiously, as they frequently do not explicitly adjust for the competing risk of non-cardiovascular mortality or the cumulative burden of treatment-emergent adverse events [15,16]. Nonetheless, this emphasis on aggregate benefit aligns perfectly with the ACC/AHA's decision to define hypertension at lower diagnostic thresholds and to mandate a universal, intensive <130/80 mm Hg treatment target for a much broader segment of the adult population [1,5].

Notably, to support this population-level risk strategy, the 2025 ACC/AHA guideline recommends using the newly developed PREVENT risk equations, which incorporate heart failure and kidney disease risk factors, to guide the initiation of pharmacotherapy in Stage 1 hypertension, replacing the previously used Pooled Cohort Equations to more aggressively capture patients who might benefit from earlier intervention [1].

Safety and tolerability considerations remain the central point of clinical divergence between the guidelines. Post hoc analyses from SPRINT, which quantified "individualized net benefit," demonstrate that the balance of risk and benefit varies substantially based on baseline cardiovascular risk and frailty [15,16]. Furthermore, age-stratified evaluations of these cohorts reinforce that older adults (aged ≥75 years) still derive significant absolute cardiovascular benefit from intensive targets, emphasizing that chronological age alone should not preclude intensive management if tolerability allows [22]. Consistent with this, long-term data from the STEP trial confirmed sustained cardiovascular benefits in older adults but also showed a persistently higher incidence of hypotension [7,12]. Importantly, both the ESC and ACC/AHA guidelines explicitly emphasize individualized treatment and shared decision-making in response to these safety signals [1,2]. However, their structural implementation differs fundamentally. The ACC/AHA framework operates as an "opt-out" strategy, defaulting to a universal <130/80 mm Hg target but allowing shared decision-making to waive it in cases of extreme frailty or limited life expectancy [1]. In contrast, the ESC utilizes an "opt-in" approach to intensive control, proactively establishing alternative, lenient targets (e.g., the ALARA principle, targeting <140/90 mm Hg) specifically for these vulnerable populations upfront [2].

Methodological issues, particularly blood pressure measurement, further explain this divergence. While analyses of SPRINT measurement protocols demonstrated that clinical outcomes were consistent regardless of whether staff were attended or unattended, the critical factor ensuring accuracy was strict adherence to a standardized rest protocol [21]. The ACC/AHA leverages this data to mandate the use of standardized measurements to achieve its intensive targets safely [1]. The ESC, however, explicitly cites the widespread failure to implement such trial-level measurement rigor in routine practice as a primary justification for its more cautious stance on a 120-129 mm Hg target [2].

Finally, international benchmarks such as the NICE guidelines highlight the broader implications of these thresholds [4]. Indeed, trial investigators and guideline committees acknowledge that intensive lowering strategies require frequent clinic visits and adherence strategies that can be challenging to implement in resource-constrained settings [1,2,7]. Thus, the lack of consensus on a single universal target reflects not only divergent clinical interpretations of net benefit, but explicit differences in how guidelines account for the practical realities, economic constraints, and measurement limitations inherent in global healthcare implementation [1,2,4].

## Conclusions

The divergence between current European and North American hypertension guidelines regarding blood pressure targets reflects a fundamental tension between maximizing population-level cardiovascular risk reduction and ensuring individual patient safety. Lower systolic blood pressure targets may offer meaningful benefit in selected patients, yet this approach requires careful consideration of tolerability and clinical context, particularly in older adults and those with complex comorbidities. The evolution of guideline recommendations suggests a shift away from rigid, one-size-fits-all numbers and toward frameworks that integrate functional assessment, cardiovascular risk stratification, and patient preference. Future management strategies are likely to depend less on debating a single universal number and more on implementing individualized treatment approaches that tailor therapy intensity to patient-specific characteristics. Ultimately, the optimal blood pressure target is one that achieves sustained risk reduction that can be maintained safely and practically for the individual patient over the long term.
